# Identifying Promising Immunomodulators for Type 1 Diabetes (T1D) and Islet Transplantation

**DOI:** 10.1155/jdr/5151171

**Published:** 2024-12-20

**Authors:** Nida Ajmal, Maislin C. Bogart, Palwasha Khan, Ibiagbani M. Max-Harry, Amber M. Healy, Craig S. Nunemaker

**Affiliations:** ^1^Department of Biomedical Sciences, Heritage College of Osteopathic Medicine, Ohio University, Athens, Ohio, USA; ^2^Translational Biomedical Sciences Graduate Program, Ohio University, Athens, Ohio, USA; ^3^Honors Tutorial College, Ohio University, Athens, Ohio, USA; ^4^Molecular and Cellular Biology Graduate Program, Ohio University, Athens, Ohio, USA; ^5^Department of Specialty Medicine, Ohio University, Athens, Ohio, USA

**Keywords:** autoimmune, immunomodulators, pancreatic beta cell, Type 1 diabetes

## Abstract

Type 1 diabetes (T1D) is an autoimmune chronic disorder that damages beta cells in the pancreatic islets of Langerhans and results in hyperglycemia due to the loss of insulin. Exogenous insulin therapy can save lives but does not stop disease progression. Thus, an effective therapy may require beta cell restoration and suppression of the autoimmune response. However, currently, there are no treatment options available that can reverse T1D. Within the National Clinical Trial (NCT) database, a majority of over 3000 trials to treat T1D are devoted to insulin therapy. This review focuses on noninsulin pharmacological therapies, specifically immunomodulators. Many investigational new drugs fall under this category, such as the recently FDA-approved CD3 monoclonal antibody teplizumab to delay the onset of T1D. In total, we identified 39 different immunomodulatory investigational drugs. FDA-approved teplizumab for Stage 2 T1D is discussed along with other immunomodulators that have been tested in Phase 3 clinical trials or higher, including otelixizumab (another anti-CD3 monoclonal antibody), daclizumab (an anti-CD25 monoclonal antibody), ladarixin (CXCR1/2 inhibitor), and antithymocyte globulin (ATG). Immunomodulators also play roles in islet transplantation and cellular therapies like FDA-approved Lantidra. Several immunomodulators involved in Phase 3 clinical studies of islet transplantation are also discussed, including alemtuzumab, basiliximab, etanercept, and reparixin, some already FDA-approved for other uses. These include alemtuzumab, basiliximab, etanercept, and reparixin, some of which have been FDA-approved for other uses. This review provides background, mechanism of action, results of completed trials, and adverse effects as well as details regarding ongoing clinical trials for each of these immunomodulators.

**Trial Registration:** ClinicalTrials.gov identifier: NCT03875729, NCT01030861, NCT00129259, NCT00385697, NCT01280682; NCT03929601, NCT04598893, NCT05757713, NCT00678886, NCT01123083, NCT00064714, NCT00468117, NCT04628481, NCT01106157, NCT02215200, NCT00331162, NCT00679042, NCT01220856, NCT01817959

## 1. Introduction

Type 1 diabetes (T1D) is a chronic autoimmune disorder that occurs due to the destruction of beta cells present in the islets of Langerhans of the pancreas. Autoreactive T lymphocytes are responsible for the destruction of insulin (INS)-producing beta cells, which results in the severe impairment of blood glucose regulation [1]. Individuals with T1D are also susceptible to other autoimmune conditions like celiac disease, vitiligo, Addison's disease, and thyroid autoimmunity presenting as either Hashimoto's thyroiditis or Graves' disease [2–4]. A study conducted using the most recent evidentiary and analytical framework from the Global Burden of Disease (GBD), injuries, and risk factors study revealed that estimated deaths due to diabetes in individuals younger than 15 years were from T1D, and the age-standardized global prevalence of T1D is projected to rise by 23.9% from 2021 to 2025 [5].

There is no cure available that prevents the destruction of beta cells. Presently, T1D-affected individuals require exogenous INS administration either through subcutaneous injections or by an INS pump, usually integrated with a continuous glucose monitoring system as well [6]. Lack of INS, being a hallmark of T1D, is a lifelong endeavor. This method of managing T1D is complicated and costly. While INS therapy and associated technologies have evolved since INS's original discovery and use, there is a need for therapy that prevents beta cell loss and maintains endogenous INS production to help regulate blood glucose levels. This review focuses on the use of immunomodulators that aim to prevent the immune response against beta cells.

### 1.1. Description of Search Parameters, Results, and Choices Made for Exclusion or Inclusion

**Figure 1:**
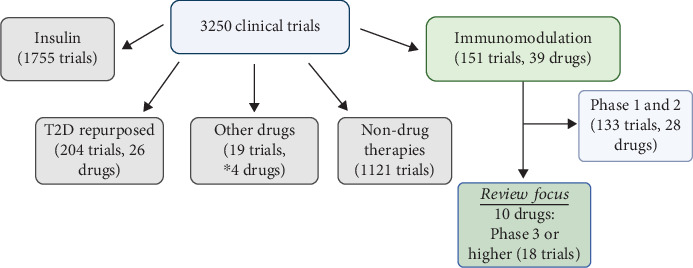
Methodology for choosing clinical trials focused on immunomodulators. Using the search term “Type 1 Diabetes Mellitus” as a disease/condition, 3250 clinical trials were identified and subdivided into categories (shown as separate boxes) based on the type of therapeutic intervention: insulin-based therapy (1755 trials), Type 2 diabetes medications (204 trials), other drugs (19 trials related to four unique drugs with potentially direct effects on beta cells that were highlighted in another review (⁣^∗^* Source:* Ajmal et al. [7])), and immunomodulators (151 trials). Among immunomodulators, only 11 drugs reached Phase 3 or higher in clinical trials related to Type 1 diabetes or organ/islet transplantation (18 trials). One of these 11 immunomodulatory drugs, rituximab, qualified for Phase 3 status, but all trials were either terminated or have not reported any data (NCT01280682; NCT03929601). The remaining 10 drugs formed the focus of this review. Another 1121 trials were listed as “nondrug therapies” because they did not fit into any category but focused on glucose monitoring systems, dietary or educational interventions, behavioral modifications, or other nondrug-based therapeutic interventions (created with Biorender.com).

ClinicalTrials.gov was searched for drugs associated with the treatment of T1D using the single search term “type 1 diabetes” as a condition. As shown in Figure 1, there were 3250 hits. This list was filtered by excluding trials that primarily studied INS, INS technologies, or INS delivery (1755 trials) and by excluding “nondrug therapies” that focused on glucose monitoring systems, dietary or educational interventions, behavioral modifications, or other nondrug-based therapeutic interventions (1121 trials). The remaining trials were binned into the following categories (see Figure 1): Type 2 diabetes medications (204 trials), immunomodulators (151 trials), and other drugs (19 trials related to four unique drugs that were the focus of a separate review [7]). The focus of this review is on the 151 clinical trials involving 39 different drugs classified as immunomodulators. This review is confined to 10 drugs that included at least one trial at Phase 3 or higher either for treatment of T1D (or at high risk for T1D) or in a pancreas/islet transplant setting.

## 2. Immunomodulators for the Treatment of T1D

### 2.1. Pathophysiology of T1D

T1D is considered an autoimmune disorder because of the presence of islet-cell autoantigens including those that bind to components of INS secretory granules such as INS, proinsulin, glutamic acid decarboxylase 65 (GAD65), glutamic acid decarboxylase 67 (GAD67), the tyrosine phosphatase islet antigen 2 (IA-2), and zinc transporter 8 (ZnT8) [1, 8–10]. Further, the appearance of these autoantigens is associated with higher T1D risk [8–10]. The autoantigens are presented on antigen-presenting cells by human leukocyte antigen (HLA) major histocompatibility complexes (MHCs) I and II to autoreactive T cells, which stimulate other cells of the immune system to produce islet cell autoantibodies (ICAs) and secrete cytokines, creating a vicious cycle of beta cell destruction [8]. Despite this knowledge, up to 15% of cases of T1D are diagnosed with no detectable ICAs, indicating that autoantibodies are an imperfect means of diagnosis [11] and that our current understanding of the pathophysiology of T1D is incomplete. The risk of developing T1D can be identified and measured, and the rate of progression to symptomatic T1D can be projected accurately. The disease is characterized by several stages: Stage 1 involves individuals with two or more islet autoantibodies who are normoglycemic; Stage 2 includes those with two or more islet autoantibodies who have glucose intolerance due to the loss of functional beta cell mass; and Stage 3 consists of typical clinical symptoms and signs of diabetes, such as polyuria, polydipsia, weight loss, fatigue, and diabetic ketoacidosis [12]. Diagnosis of T1D typically occurs at Stage 3.

Evidence that there is a genetic component to the pathogenesis of this disease includes the increased risk of developing T1D if a family member is affected and with certain alleles at the *HLA DR*, *HLA DQ* Class II, *INS*, and *PTPN22* (Protein Tyrosine Phosphatase Nonreceptor Type 22) loci [10, 13]. However, genetics cannot fully explain the initiation of T1D pathology because only 5% or fewer of people with high-risk haplotypes develop T1D, and monozygotic twins do not always both develop T1D [8, 13]. Therefore, environmental triggering factors must play a role. Evidence has been presented that epigenetic factors, DNA methylation, or exposure to INS in utero, for example, could determine if T1D develops in an individual [8]. Additionally, environmental factors such as viruses, season (people living in colder climate regions are at higher risk of T1D), geographic location, vitamin D deficiency, and diet have been suggested to contribute to T1D incidence [8, 13]. However, a causative link between these factors and T1D development has not been established. Overall, the understanding of the pathogenesis of T1D is incomplete, and continued research is required to present potential therapeutic targets to delay or prevent the onset of T1D.

The destruction of beta cells in T1D occurs through an immune response, which triggers insulitis by either activation of endogenous signals or by the exogenous response involving viral attacks. Immune cells are recruited when interferons and other cytokines are released from islet beta cells [14]. Macrophages, which are among the main sources of tumor necrosis factor (TNF), respond and activate proapoptotic natural killer (NK) cells. This inflammatory response progresses to infiltration of islets by cytotoxic T cells that include CD20+ B cells, CD4+ T cells, and CD68+ macrophages [15]. IL-1beta and interferon (IFN)-gamma initiate proinflammatory pathways that disrupt beta cell function, metabolic activity, and INS granule synthesis [16]. The onset of symptom presentation occurs when almost 75% of beta cells have been destroyed as a result of the immune response [17]. The resulting condition is an INS deficiency in the body leading to hyperglycemia and other complications. Thus, immunomodulation is a logical strategy to prevent the loss of beta cells and halt or even reverse the disease process.

### 2.2. Immunomodulatory Therapies

Immune-focused therapies are designed to protect beta cells from the immune system and prevent the progression of T1D. One of the earliest indications that immunomodulators could be therapeutic in T1D was a pilot study that used cyclosporine (a calcineurin inhibitor that inhibits T-helper cells and is used commonly as an immunosuppressor in organ transplantations) in participants with new-onset T1D [18]. In this pivotal study, participants within 6 weeks of diagnosis who were treated with cyclosporine for 2–12 months demonstrated improved C-peptide levels and reduced evidence of beta cell autoantibodies [18]. Although our understanding of the pathology of T1D has improved in the 40 years since this study, clinical trials of cyclosporine continue, including a current Phase 2 trial of cyclosporine followed by low-dose interleukin-2 (IL-2) in new-onset T1D.

The focus of this review is on immunomodulators that either deactivate or eliminate pathogenic cells or enhance regulatory T cells (Tregs). Tregs, critical regulators of peripheral immune tolerance, play a key role in maintaining immune homeostasis, and insufficiency in Tregs can lead to T1D. IL-2, a cytokine secreted by Teff cells (T effector cells), binds to the alpha chain of interleukin receptor-2 (IL-2R) receptor CD25 on Tregs, promoting their expansion and enhancing immune tolerance. However, abnormal IL-2 signaling in CD4+ T cells can downregulate the expression of FOXp3, a transcription factor essential for the development and proper function of Tregs [19, 20]. Such abnormalities in CD4+ T cells result in Treg depletion. The balance between Teff activation and Treg suppression is important in immune tolerance, whereas in T1D this balance is disrupted, resulting in hyperactive Teff cells and reduced Tregs. Monoclonal antibodies have been shown to increase the number and potentially improve the function of Tregs. These antibodies also downregulate pathogenic T cells by enhancing Tregs activity and modulating the immune response [18, 19].

**Figure 2:**
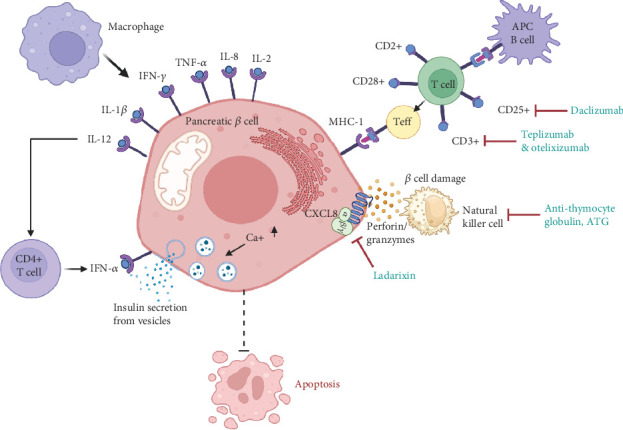
Graphical representation of the pathogenesis of T1D by immune response and how immunomodulators are involved in the prevention of T cell immune response. This immune response occurs by macrophages and natural killer cells that activate and produce proinflammatory cytokines and trigger insulitis. B cells also contribute to the pathogenesis of T1D and act as antigen-presenting cells (APCs) for antigens. B cells present antigens to autoreactive T cells and mediate pancreatic cell cytotoxicity by major histocompatibility complex. Immunomodulators like daclizumab, teplizumab, otelixizumab, ATG, and ladarixin modulate T cells and natural killer cells and prevent pancreatic beta cell immune response and apoptosis (created with Biorender.com).

Figure 2 illustrates how the immune response of both macrophages and autoreactive T cells is involved in causing beta cell dysfunction. This figure also addresses how immunomodulators bind to T cells and are involved in regulating immune response. These therapeutics aim to prevent beta cell destruction, restore beta cell mass, and/or enhance INS secretion, thereby attempting to treat and prevent this disease. Prevention studies have focused on establishing self-tolerance among those at risk of development [21]. As mentioned previously, we have limited our focus to drugs that have reached at least Phase 3 status in the National Clinical Trials database. These are listed in Table 1 and discussed individually in the following section.

### 2.3. Teplizumab

Anti-CD3 monoclonal antibodies have been hypothesized to prevent and treat T1D by binding to the surface of T cells involved in antigen recognition to form CD3-epsilon, which is a part of the T cell receptor (TCR) complex. The TCR complex is composed of alpha and beta chains and six CD3 molecules, among which two CD3 epsilon chains are anchored in the cell membrane. This complex is known to be involved in the inhibition of cytotoxic T cells [22]. This interaction leads to the phosphorylation of six CD3 chain molecules, resulting in a change in cell behavior and effector function, such as T cell cytotoxicity and cytokine secretions of TGF-beta, IFN-gamma, and TNF-alpha [23]. Anti-CD3 monoclonal antibodies inhibit T cell cytotoxicity and also work as T cell mitogens when used in lower concentrations [22, 24]. At higher concentrations, anti-CD3 antibodies reduce T cell cytotoxicity by disrupting interactions between TCR complexes and peptide-MHC molecules, leading to the development of therapeutic strategies [25].

Teplizumab is the first humanized anti-CD3 monoclonal antibody and non-INS–based pharmacologic approved to treat T1D since pramlintide. A landmark clinical trial demonstrated that teplizumab delayed T1D progression for 2 years in individuals at high risk after a 2-week treatment course [26]. Teplizumab has also been shown to delay the need for exogenous INS for up to 3 years and maintain beta cell function. Additionally, it increased C-peptide levels, which helps reduce complications associated with T1D [27]. In an earlier Phase 3 clinical trial of teplizumab, participants who received teplizumab developed antidrug antibodies without affecting drug efficacy or causing tolerance issues [28]. Following key clinical trials, teplizumab was approved by the FDA as of November 17, 2022, and sold by the trade name Tzield [28, 29].

A subsequent Phase 3 trial examined whether two courses of teplizumab can prevent the loss of beta cells in recent-onset T1D. The trial findings relied on two clinical end points: the primary end point focused on change from the baseline in beta cell function measured through C-peptide levels, while the secondary endpoint focused on INS use, glycemic control, *HbA1c* levels, and hypoglycemia. Results showed that teplizumab delayed the onset of Stage 3 T1D in patients aged 8 years or older with Stage 2 T1D [30]. Significant improvements in beta cell function were also present, though no improvements were observed in the secondary endpoints. Episodes of adverse effects included headaches, gastrointestinal issues, lymphopenia, and mild cytokine release syndrome (CRS) [30].

Several additional clinical trials for teplizumab in the treatment of T1D are underway. A Phase 3 clinical trial is examining long-term safety, while a Phase 4 clinical trial will investigate the use of teplizumab among pediatric participants with Stage 2 T1D. This clinical trial is still being recruited, and the outcomes will determine the pharmacokinetics, safety, and tolerability of teplizumab after its completion.

In addition to the abovementioned clinical trials, a meta-analysis study conducted for eight different clinical trials on teplizumab's safety and efficacy showed that teplizumab therapy was associated with reduced INS use and an increase in C-peptide area under the curve (AUC) levels; however, teplizumab did not significantly affect HbA1c [31]. The meta-analysis also highlighted teplizumab-associated adverse effects such as lymphopenia, skin, and subcutaneous tissue disorders [31]. Another meta-analysis addressed preclinical, Phase 2 and 3 studies on teplizumab and similar antibody therapy. The results demonstrated the effectiveness of teplizumab among individuals with a high risk of developing T1D and less effective in newly diagnosed T1D participants [32]. These findings suggest teplizumab's efficacy in delaying the progression of T1D and still requires a better immunotherapeutic approach which can serve best in the treatment of immune-derived T1D.

Anti-CD3 therapies are found to increase C-peptide response, in other words, to improve pancreatic beta cell function while offering manageable adverse effects, per many studies conducted. However, these immunotherapies require careful screening of more serious adverse effects such as lymphopenia, viral infections, and CRS. Additionally, these therapies also necessitate long-term studies to be conducted to test immunotherapy durability and dosage frequency. Studies with longer follow-ups are needed to evaluate the efficacy, durability of therapeutic response, and safety of these immunotherapies in T1D due to the risk of CRS and reactivation of viral infections. Moreover, the cost of these therapies is a significant limitation that needs to be addressed.

Despite these limitations, novel treatments like teplizumab have revolutionized T1D therapeutics by inducing chronic immunosuppression targeting T cell receptors, the major player in T1D progression, to treat T1D before it progresses to hyperglycemia. Although this delay in disease onset could be considered modest (1–2 years), the proof-of-concept now exists, such that future immunotherapy therapies could extend this window.

### 2.4. Otelixizumab (ChAgly CD3)

Another anti-CD3 monoclonal antibody in clinical trials is known as otelixizumab (ChAgly CD3), which also targets CD3 T cells. This anti-CD3 was first used with cyclosporine, but it was no longer effective after the withdrawal of cyclosporine [18, 33, 34]. Otelixizumab is a humanized, nonmitogenic, Fc-modified, anti-CD3 monoclonal antibody that has limited Fc receptor binding sites [35]. Otelixizumab is also known as TRX4 [36], and it works by binding on the receptor present in T cells such as CD3, which is involved in T cell signaling. This monoclonal antibody consists of two heavy chains and two light chains. The heavy chains are humanized gamma-1 chain from rats, whereas light chains are chimeric human chains. Otelixizumab inhibits the function of T cells by inducing Treg pathways and promoting active immune tolerance that eventually inhibits the autoimmune reactivity of a disease. Genetically modified otelixizumab helps in reducing the inflammatory adverse effects by removing the glycosylation site in the Fc domain and results in the depletion of Fc receptor binding [37]. Studies also showed that otelixizumab downregulates T cells that are pathogenic, upregulates T regulatory cells, and reduces the immune response in T1D condition [38].

In a preclinical study in mice, otelixizumab at an 8-*μ*g dose showed a 53% reduction in the severity of T1D and modulated CD3 T cell for up to 30% which provided long-term success in reducing T1D [39]. A study conducted on otelixizumab showed that a dosage of 3.1 mg was well tolerated in T1D-affected individuals, whereas it did not show effective results in the preservation of C-peptide levels and in metabolic control [40]. Efficacy studies on otelixizumab showed different risk and benefit results when the same dosage of 48 mg was tested. It is thus recommended to consider the relative dosages of otelixizumab while conducting clinical trials [40–42].

A Phase 3 clinical trial was conducted to examine if otelixizumab preserves beta cell function and facilitates long-term glycemic control. This multicentered, randomized, and placebo-controlled trial enrolled 281 patients, and otelixizumab was administered over 8 days and compared with placebo control. C-peptide levels from a 2-h mixed meal tolerance were measured at month 12. The conducted clinical trial found that otelixizumab was well tolerated by patients with new-onset T1D, but it was not found to be effective in restoring beta cells, C-peptide, and other aspects of metabolic control. The clinical trial also reported adverse events such as headache, fever, rash, nausea, and side effects related to anti-CD3 antibodies [40]. Another Phase 3 clinical trial was conducted in which otelixizumab was given to 80 new-onset T1D patients for six consecutive days. Enrolled subjects were followed for 18 months to monitor INS needs and residual beta cell function. The recent findings of this trial showed better residual beta cell function and INS independence which were maintained among the patients who were given otelixizumab compared to the placebo group for up to 18 months, thus representing otelixizumab as a short-term treatment for T1D patients. The reported adverse events were headache, gastrointestinal symptoms, arthralgia, and myalgia [35].

Although humanized anti-CD3 has been shown to be effective in regulating the baseline plasma C-peptide level among stage 3 T1D individuals, adverse effects have raised concerns. A placebo-controlled trial on otelixizumab restored beta cell function for 18 months among new-onset T1D-affected individuals; however, 75% of otelixizumab-treated participants developed symptoms associated with acute mononucleosis and CRS [35]. Besides that, reported side effects caused by otelixizumab are headache, fever, nausea, and rash. Seventy-five percent of enrolled subjects showed Epstein–Barr virus (EBV) reactivation and had symptomatic disease, and a lower dosage is recommended that will help prevent EBV reactivation and T1D severity [40].

### 2.5. Daclizumab

Daclizumab is an anti-CD25 humanized monoclonal antibody that reduces the consumption of IL-2 by effector T cells, inhibiting T cell activation, expansion, and survival [43]. Daclizumab is used for other T cell-mediated immune conditions like uveitis and multiple sclerosis [44]. Daclizumab inhibits CD25 activity by binding it to the alpha subunit of the IL-2 receptor that is present in lymphocytes. IL-2 binding and progression of T lymphocytes are inhibited by anti-CD25, also termed daclizumab [45].

Daclizumab was investigated in a Phase 2 randomized clinical trial where it was used as an immunosuppressive in combination with exenatide, a glucagon-like peptide-1 (GLP-1) agonist, used to stimulate beta cell recovery and regeneration. Twenty subjects with long-standing T1D were enrolled, and after achieving optimal glucose control, 16 subjects in total were selected to receive the exenatide with or without daclizumab. Endogenous INS production was monitored by measuring C-peptide levels. The results showed that in long-standing T1D, surviving beta cells secrete INS in a physiological manner, but the combination of intensive INS therapy with GLP-1 agonist and immunosuppressive drugs was not effective in improving beta cell function. Moreover, this trial showed delayed gastric emptying and suppressed endogenous incretin levels, but no increase in C-peptide levels was observed [46]. More combination therapies are recommended for residual beta cell function in younger patients and early T1D-diagnosed individuals [46]. A Phase 3 trial has been conducted among T1D recipients of kidney transplants to assess the benefits of islet transplantation. This clinical trial allowed participants to remain on immunosuppressive therapy that included daclizumab with other immunomodulators. The outcomes of this trial improved HbA1c ≤ to 6.5%, a reduction in INS requirement, whereas no hypoglycemic events were found among participants. This trial has been completed with no published results.

Although these clinical trials showed improved HbA1c and beta cell survival, there were some adverse effects reported after the use of daclizumab. Gastrointestinal infections, reactivation of infections that were previously reported, neutropenia, leukopenia, elevated liver enzymes, and major hypoglycemic events were observed [45]. It has been recommended that daclizumab undergo additional dose optimization because a lower dose is not found to be effective in activating effector cells in the pancreas, and a higher dose showed greater therapeutic effect but with adverse events [45].

### 2.6. Ladarixin

Chemokine ligand 8 (CXCL8) plays a significant role in the pathogenesis of T1D and is involved in the recruitment and trafficking of neutrophils via receptors such as CXCR1/CXCR2. Due to proinflammatory cytokines, pancreatic islets produce and secrete CXCL8 found to be responsible for the pathogenesis of T1D [47–51]. Moreover, chemokine receptors like CXCR1/2 are found to be elevated in the blood of both mice and humans with T1D [52–54]. CXCR1/2 inhibition is effective in the prevention of inflammatory damage during the development of diabetes. One of the potent CXCL8 inhibitors is laddering, which reduces neutrophils, lymphocytes B-1a, and plasmacytoid dendritic cells that play a crucial role in the progression of T1D [55]. A study of NOD mice investigated CXCL8 blockade and found that ladarixin was effective in the prevention and reversal of diabetes. Blocking CXCL8 is linked with insulitis and further modifies leukocyte distribution in the organs such as blood, bone marrow, lymph nodes, and spleen. Ladarixin in combination with reparixin reduced CXCR2+ myeloid cells the most among leukocyte subpopulations. This study in mice further concluded that CXCL8 and CXCR1/2 are major chemokine factors in diabetes etiology [56].

A preclinical study found that inhibitors like ladarixin effectively inhibit IL-8 signaling and CXCR1/2 receptors, which then prevent trafficking and recruitment of neutrophils during T1D [57]. These studies led to the initiation of clinical trials in which ladarixin showed a short-term inhibition of CXCL8 receptors, but it was not found to be effective in restoring beta cell function in newly diagnosed T1D [58]. It is also suggested that ladarixin in combination with other therapies would be beneficial in inhibiting IL-8 signaling to treat T1D. A Phase 3 clinical trial is in the recruitment stage to test if oral ladarixin is effective in the preservation of beta cell function or in delaying the progression of T1D among adults and adolescents. No results of this trial have been released to date.

Regarding adverse effects, an early-phase clinical trial found ladarixin to be safe and well-tolerated by the enrolled participants. Treatment-emergent serious adverse events (TESAEs) were mild and included infections, gastrointestinal disorders, dyspepsia, and headaches [58]. Due to the complex nature of CXCL8 receptors and IL-8, further investigation is warranted to clarify the long-term therapeutic effects of ladarixin alone and in combination with other immunosuppressants [58].

### 2.7. Antithymocyte Globulin (ATG)

ATG is a long-standing immunosuppressant used to prevent transplant rejection by reducing T lymphocytes, NK cells, and dendritic cells [59, 60]. ATG is also used to prevent and treat severe aplastic anemia and other autoimmune diseases, and it has shown potential promise in the treatment of T1D. ATG also modulates B lymphocytes and other cells involved in the immune response [60]. The immunosuppressive activity of ATG infusion depends primarily on T lymphocytes and results in peripheral immunosuppression which depletes peripheral lymphocytes after 24 h of exposure [61]. The depletion of lymphocytes occurs by ATG infusion through mechanisms such as apoptosis, complement depletion analysis, and antibody-dependent cytotoxicity that have been investigated in vitro. ATG downregulates different cell surface epitopes such as CD3, CD-7, CD-8, CD-19, CD20, CD-32, and CD-28 [61].

A preclinical study showed that the administration of low-dose murine ATG and granulocyte colony-stimulating factor (GCSF) significantly reversed T1D in NOD mice [62]. ATG and GCSF synergy seemed promising in the reduction of the immune response, as ATG reduced T lymphocytes and GCSF promoted Tregs [63]. In another preclinical study, murine ATG reduced the severity of T1D in NOD mice in an age-dependent manner, which suggested that ATG-mediated immunoregulation is effective in early stages or in late prediabetes (12 weeks) stages [64]. Murine ATG therapy in an age-dependent fashion reversed pancreas insulitis, improved glycemic control, and caused a rapid shift in the increase of antigen-presenting cells in the spleen and pancreatic lymph nodes [64].

Several clinical trials have also been conducted to study the potential therapeutic effects of ATG against T1D. A randomized, single-blinded, placebo-controlled pilot study involved 25 subjects receiving ATG/GCSF treatment compared with placebo control. The change in AUC levels following a 2-h mixed meal tolerance was monitored for up to 1 year. The trial showed a borderline improvement in beta cell function relative to placebo (*p* = 0.050) as measured by C-peptide. Hypoglycemic events, headache, fatigue, respiratory infection, and anemia were observed as adverse events after drug administration [65]. ATG alone in the Phase 2 clinical trial was found effective at low doses, restoring C-peptide AUC with a mixed meal tolerance test (MMTT) along with beta cell preservation [66]. The study also found that ATG at low doses is effective in suppressing the immune response and reducing the number of total lymphocytes and CD4+ T cells while preserving CD-8+ T cells, beneficially reducing the CD4+/CD-8+ ratio [66]. A follow-up study conducted 5 years after treatment, however, showed no significant difference in AUC for C-peptide when comparing ATG/GCSF to placebo (*p* = 0.41) [67]. The mechanistic pathway of ATG at low dose is still being studied to elucidate the immunologic associations.

A Phase 3 clinical trial has been conducted in which the safety and efficacy of islet transplantation were investigated along with immunosuppressive medications among T1D individuals who were experiencing severe hypoglycemic conditions and hypoglycemic unawareness. The immunosuppressive drugs including ATG were found effective in maintaining health-related quality of life. Also, this trial showed improvements in severe hypoglycemic episodes of the enrolled participants along with improved glycemic control after a year postislet transplantation [68]. Another Phase 3 trial involved using immunosuppressives in kidney transplant participants who also received islet transplantation. These participants followed a postsurgery regimen of immunosuppressive drugs that included ATG. However, this clinical trial has no published results to date. Other drugs that have been used primarily in the transplant setting in Phase 3 trials are discussed in the following section.

The adverse effects of ATG at low dose (2.5 mg/kg) in pediatric and adult populations were found to be similar and manageable. Some of the reported adverse events are associated with CRS such as fever, headache, nausea, lymphopenia, and serum sickness. These adverse effects can be managed by slow intravenous drug infusion and premedication with hydrocortisone, heparin, corticosteroid, antihistamine, and acetaminophen (paracetamol) [61, 67, 69].

In closing immunomodulatory therapies, some other immunomodulators such as tocilizumab, hrIFN-alpha, rituximab, and golimumab lack clinical approval after advancing through Phase 2 clinical trials for T1D due to the contribution of different factors. These immunomodulators often demonstrate limited, inconsistent efficacy in preserving pancreatic beta cell function. There are safety concerns including off-target–binding and the risk of long-term complications. These immunomodulators lack long-term, promising potential effects in treating T1D condition. For instance, rituximab showed adverse effects such as infusion reactions occurring within 24 h of administration. Likewise, other immunomodulators have drawbacks too due to which their clinical approval has not been made.

Preventing the autoimmune destruction of beta cells and maintaining beta cell function is crucial in treating T1D condition, but it is equally important that the body's immunity should not be compromised. Despite ongoing clinical trials for immunomodulators designed to induce immunological tolerance, these approaches face significant obstacles. One major obstacle is due to three major autoantigens such as GAD65, IA-2, and INS in T1D which makes it difficult to know whether one, two, or all three major autoantigens are required to induce immunological tolerance, and this uncertainty complicates the efforts to preserve beta cell function and INS production [70]. Also, the underlying cause of autoimmune destruction of beta cells is not well understood, which hampers immunomodulatory therapies. Once beta cell destruction is slowed or halted, beta cell mass and/or function must also be restored, which is the focus of important research that is outside the scope of this review [71].

## 3. Immunomodulators in Islet Transplantation

Another important use of immunomodulators is in islet transplantation procedures. Islet transplantation (also named islet allotransplantation or autotransplantation) is an accepted therapeutic option recommended when T1D worsens. The islet transplantation procedure involves taking islets from a healthy (deceased) donor and transplanting them to the portal vein that carries blood from the pancreas to the liver of the T1D recipient. As a result, islets begin to make INS and regulate hyperglycemic conditions. In addition, transplanted islets can also lower or remove the need to use INS injection to regulate blood glucose levels and enhance control of both hypoglycemic and hyperglycemic events [72].

With the development of new immunosuppressive medications in the mid-1980s, pancreatic transplantations increased dramatically. The success rate improved, and approximately 80% of the recipients were INS-independent after pancreas transplantation [73]. In terms of INS independence, pancreas transplantation is more effective than islet transplantation, but the procedure involved in pancreatic transplantation is complicated and may lead to thrombosis, bleeding, duodenal leaks, and results in graft loss or morbidity, whereas islet transplantation has no major complications associated during or after islet infusion [74]. In addition to the simplicity and safety of the procedure, islet transplantation allows the use of cell banking after cryopreservation and offers the advantage of a pretransplant reduction of immunogenicity and xenotransplantation [75].

Islet transplantation procedures improved gradually over the 1970s through the 1990s. In 2000, Shapiro et al. proposed advanced methods of donor selection, islet isolation, transplantation, and required posttransplantation measures using immunosuppressive regimens [76, 77]. In 2006, the Edmonton protocol was published, which showed restored endogenous INS production for up to 2 years and better glycemic controls after islet transplantation in T1D-enrolled subjects [78, 79]. Clinical trials on islet transplantation showed promising effects such as improved glycemic control and T cell depletion during immunosuppressive regimen [80, 81] and have continued to show progress in overall outcomes [79, 82].

Not every islet transplantation is successful, and the potential cause of graft loss includes recurring autoimmunity, cell death by the antagonistic environment, toxicity caused by drugs, and even hyperglycemic conditions [83]. Islet transplantation could be more effective when combined with immunosuppressants, and different procedures have shown effective results when it is combined with immunomodulators like cyclosporine, glucocorticoids, and azathioprine [84]. With the advancement of new and emerging immunomodulators, there are possibilities to make islet transplantation in combination with an immunosuppressive regimen more effective, which would help reduce diabetogenic effects and provide immune protection. Islet transplantation combined with glucocorticoid-free immunosuppressives such as daclizumab, sirolimus, and tacrolimus provided INS independence and improved metabolic control [77]. These induction therapies are expected to target already existing T cells and restrict the proliferation and development of antidonor T cells, ultimately preventing allorejection [85]. Immunosuppressive drugs like anti-CD25+ T cells restore immune regulation by the expansion of Tregs and provide a promising approach for autoimmunity and islet transplantation survival [86]. The reappearance of T cells causes graft loss, requiring prolonged intake of daclizumab (anti-CD25+), which can become ineffective over time. Therefore, the removal of CD25+ cells would be helpful in graft survival. Studies reported that preventing anti-CD25+ subsets might benefit graft survival via immune regulation [87, 88]. However, graft removal has been found to be harmful during the maintenance phase [83]. In addition to T cell inhibitors, ATG helped improve islet transplantation.

**Figure 3:**
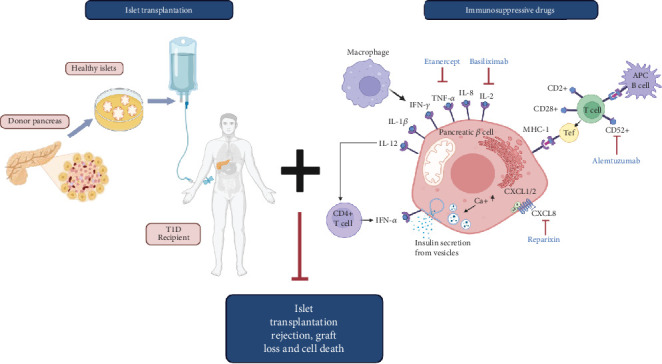
A graphical representation of basic steps involved in islet transplantation along with immunosuppressive therapy to help eliminate islet transplantation rejection, graft loss, cell toxicity, and cell death. Pancreatic islets from healthy donors are transplanted into T1D recipients, and a regimen of immunosuppressive drugs is recommended that prevents graft loss and transplantation rejection. Immunomodulators such as etanercept, basiliximab, reparixin, and alemtuzumab are some of the most common drugs that target TNF-alpha, IL-2, CD52+, and CXCL8 receptors, respectively (created with Biorender.com).

Many immunosuppressive drugs have been examined for possible use in islet transplantation settings to preserve or enhance the viability and function of islet grafts, and several compounds are currently being tested in clinical trials of islet transplantation [89–91]. The possibility of preventing the loss of islet graft can also be achieved by using high-quality islets from multiple donors, and the immune rejection can be prevented by the transplantation of mesenchymal stem cells (MSCs)/Tregs or with the use of advanced islet encapsulation techniques in addition to immunosuppressive drugs [79, 92]. Considering immunomodulators as a center of attention for this review, Figure 3 illustrates islet transplantation steps with the use of immunomodulators that help prevent graft loss and cell death. In Table 2, we list immunomodulators that have reached Phase 3 or higher in clinical testing and have been incorporated into islet transplantation trials. Each is discussed in detail below.

### 3.1. Alemtuzumab

Drugs incorporated into Phase 2 or 3 trials include alemtuzumab (Lemtrada), which is an anti-CD-52+ monoclonal antibody that has been FDA-approved to treat chronic lymphocytic leukemia. CD-52+ is a glycoprotein that is present on the cell surface [93], and anti-CD-52+ monoclonal antibody helps deplete CD-52+-expressing mature lymphocytes and repopulate immune cells, thus modulating the immune system [94]. This drug has been licensed as a treatment for highly active relapsing-remitting multiple sclerosis (RRMS) [95] and is now under study as a combination drug for islet transplantation [93]. A Phase 4 clinical trial involved testing alemtuzumab as an induction therapy in kidney and pancreas transplantation, and a comparison was made with ATG. The efficacy of alemtuzumab was evaluated in enrolled recipients of kidney and pancreas transplants. The findings identified both alemtuzumab and ATG as potential outcomes in preventing graft loss and increasing long-term patient survival with no early adverse effects upon induction. This trial suggested alemtuzumab induction in combination with other steroid-free immunosuppressive drugs results in effective graft survival but is limited by the risk of infections and secondary autoimmune disorders [96].

### 3.2. Basiliximab

Another immunomodulator known as basiliximab is a monoclonal antibody that acts as an IL-2 receptor antagonist to prevent organ rejection with other combinations of immunosuppressive drugs [97]. It binds to the IL-2R alpha on the cell surface of activated T lymphocyte to prevent the signaling of IL-2/CD25 [98], thereby preventing white blood cells from rejecting the graft. The efficacy of basiliximab is similar to ATG in lowering the risk of tissue rejection [97]. A study found that basiliximab treatment produces equivalent short-term results in those who received depleting antilymphocyte induction therapy [99]. In comparison, another study showed improved allograft success and INS independence, which concluded that an immunosuppressive regimen is effective in both the prevention of allograft rejection and in controlling the immune response [77]. Together, these studies indicate that INS independence can be achieved when an islet mass is transplanted, followed by a regimen of immunosuppressive drugs.

A Phase 3 clinical trial was conducted in which participants received up to three islet transplants followed by a regimen of a combination of immunosuppressive drugs. Basiliximab was used in place of ATG in a second and third islet transplantation infusion, only if necessary. The results showed improved glycemic control and reduced hypoglycemia. The trial found mild to adverse effects associated with the administration of basiliximab such as cytopenia, abdominal pain, serum sickness, and CRS [81]. Another Phase 3 islet transplantation clinical trial demonstrated the safety and efficacy of islet allograft with improved glycemic control in T1D. Participants received other interventions along with testing basiliximab. This trial has no published results; however, another study conducted showed that basiliximab induction therapy prevents acute rejection but increases the risk of developing a new onset of diabetes after transplantation (NODAT) [100]. The most commonly reported adverse events of basiliximab in adults are constipation, nausea, headache, hypophosphatemia, hyperkalemia, hypercholesterolemia, anemia, and hypertension [98].

### 3.3. Etanercept

Also known as recombinant solvent TNF-alpha receptor fusion protein, etanercept acts as a TNF-alpha inhibitor by binding to TNF-alpha, clearing it from circulation, and blocking its inflammatory activity. A study found that islet allograft rejection occurs because of TNF-alpha, and a blockade of TNF-alpha in combination with IL-1beta (anakinra) can improve islet engraftment, which also results in INS independence when followed with a regimen of immunosuppressive drugs [89]. Etanercept showed a significant improvement in pancreatic islet graft survival [101]. It has been previously reported that etanercept induces a higher rate of INS independence in 240 islet allograft recipients with fewer adverse events at suitable concentrations [101, 102]. Etanercept has been tested for T1D and beta cell survival and has also been tested to prevent islet allograft rejection [93]. A study found that etanercept facilitates islet allograft function and helps INS independence with a few islets. Moreover, this study also found that the anti-inflammatory effect of etanercept also helped in early islet function and improved engraftment. However, this study also mentioned that etanercept has side effects such as increased risk of developing multiple sclerosis, seizures, risk of lymphoma, blood disorders, and reaction on injection sites, which could occur with prolonged use as required to treat autoimmune disorders [103]. A Phase 3 clinical trial has been completed where etanercept, along with other immunomodulators, showed improved engrafted islet beta cell mass in T1D participants who received islet transplantation [81]. Another Phase 3 clinical trial examined etanercept and other immunosuppressive drugs to assess the benefit of islet transplantation among T1D participants who received kidney transplants. This trial has been completed, but no results have been published.

### 3.4. Reparixin

Reparixin is an inhibitor of CXCR1/2 chemokine receptors [104], which are G-protein-coupled receptors on neutrophils. IL-8 (CXCL8) activates these receptors to induce T cell and NK-cell migration at the inflammation site. A study in mice showed that reparixin deactivated the receptors involved in CXCL8 signaling by inducing conformation changes that ultimately inhibit proinflammatory cytokine production and leukocyte infiltration [52, 105–109]. A preclinical study found that reparixin, in combination with CTLA-4 Ig, downregulated T cell recruitment and reduced neutrophil activation after islet transplantation for a more extended period. Another study found that the CXCR1/2-mediated pathway is a regulator of islet damage and that reparixin improved the efficacy of islet transplantation [50]. In a study of mice, CXCR1/2 blockade resulted in full graft survival after transplantation. This study also found reversal and prevention of T1D in mice and reduced autoinflammation [56]. Based on promising preclinical results, a Phase 2 clinical trial was initiated to test the efficacy of reparixin in preventing islet transplantation failure among T1D patients. However, no results could be found for this trial. A Phase 3 multicentered, randomized, double-blind clinical trial was conducted on allotransplant recipients to study the effect of reparixin. AUC, INS independence, and glycemic controls were monitored between placebo and reparixin-treated groups. The findings of the study showed no significant difference in the C-peptide levels of the placebo and reparixin groups, whereas INS independence was observed for up to 1 year in patients who received reparixin after islet transplantation [110]. In short, the preclinical and clinical trials showed significant short- and long-term islet graft survival results among T1D patients [67]. However, a longer follow-up is required to fully understand the benefit of islet transplantation to help treat T1D with a regimen of immunomodulators [111]. Reparixin showed no prolonged adverse events upon administration. Most side effects reported throughout the clinical trial were related to blood and lymphatic system disorders, metabolic disorders, musculoskeletal and connective tissue, and nervous system disorders [110].

In closing, several benefits and risks are associated with all immunotherapies available for T1D management. Pancreas and islet transplantation offers benefits such as normal glucose homeostasis recovery and high patient survival rates achievement but also carries limitations such as costly major surgery approaches with a high incidence of complications, specialized expertise requirements, and lifelong immunosuppression. Advanced immunotherapeutics such as an antibody (teplizumab, otelixizumab, etc.) and antigen-based immunotherapies (autoantigenic peptides and proteins antigen-presenting cells (APC), etc.) offer potential for targeted, individualized approaches, especially in new disease onset and preventive settings. They also face challenges like identifying the best treatment for different stages of the disease, understanding patients categorization for the right treatment, and conducting enough clinical trials to optimize treatment timing and balance between benefits and limitations.

### 3.5. Lantidra (Donislecel): Islet Cell Therapy

Although not an immunomodulator, we note that the FDA-approved donislecel, under the brand name Lantidra, will be the first allogenic islet-cell therapy for T1D treatment in June 2023. Donislecel involves the infusion of dispersed donor islet cells instead of grafting intact human islets. Clinical trials provided evidence that donislecel is a successful therapy for INS secretion from infused pancreatic beta cells [112, 113]. There are also adverse events reported due to the infusion of this therapy including fatigue, anemia, nausea, diarrhea, and abdominal pain [112]. As with islet transplantation, immunosuppressive adjunctive therapy is suggested for donislecel to prevent cell death due to the immune response that often causes numerous pathologies. The efficacy of donislecel may improve by optimizing cell harvest procedures and preventing tissue rejection with an effective immunosuppressive regimen [112]. Although early clinical trials appear promising, donislecel shares similar concerns as islet transplantation regarding adverse effects and tissue rejection. Therefore, immunomodulators reviewed here are still necessary for donislecel to meet its full therapeutic potential.

## 4. Conclusion

While INS is unquestionably the most effective therapy for T1D developed in the last 100 years, our enhanced understanding of the role of the immune system and the beta cell in its own demise has opened additional therapeutic avenues. T1D contributes substantially to disability-adjusted life years (DALYs) particularly due to associated complications. The global burden of T1D and the growing incidence rate required an urgent demand to develop novel therapeutic interventions. Various monoclonal antibody therapies have been developed that modulate the activity of Teff cells, T regulatory cells, and other immunological processes. We have highlighted several drugs in the pipeline that have shown varying degrees of success in delaying the progression of T1D and preserving functional beta cells. According to PubMed and clinicalTrial.gov searches on Phase 3 clinical trials, some emerging immunomodulators that might progress to Phase 3 trials are also under study. Phase 2 clinical trials have been initiated or completed for anti-IL-6 receptor tocilizumab, human recombinant interferon-alpha (hrIFN-alpha), anti-CD20 monoclonal antibody rituximab, and TNF-alpha inhibitor golimumab. To date, the drugs listed above have not been approved for clinical use. Additional investigational studies are required on their therapeutic effects.

For any therapy to successfully reverse T1D, two objectives must be met: (1) restoration of INS secretory capacity and (2) prevention of autoimmune destruction of pancreatic beta cells. Islet transplantation and cell-based therapies like donislecel address the first objective, but without addressing autoimmunity, these approaches fail to meet their ultimate potential. Likewise, immunotherapies may delay T1D progression, but without restoring lost beta cells, full recovery of INS capacity is unlikely. The approval of teplizumab in the treatment of Stage 2 T1D has set a new bar for future immunomodulatory therapies, which, in conjunction with other INS-restoring therapies, may 1-day halt or even reverse the progression of T1D. The clinical implications of these advances are vast, as they offer new opportunities to personalize treatment regimens, reducing dependency on lifelong INS therapy, and improving patient outcomes. Further research is critical to address gaps in knowledge of immune regulation and to develop more effective therapies for long-term management and potential reversal of T1D.

## Figures and Tables

**Table 1:** Investigational new drugs with immunomodulatory properties. Created with Biorender.com.

**Drug name(s)**	**Target**	**Side effects**	**NCT number(s)**	**Published results**	**Key findings**
Teplizumab (Tzield)	Anti-CD3 monoclonal antibody	• Headache • Gastrointestinal issues • Lymphopenia • Mild cytokine release syndrome (CRS)	NCT00385697 NCT04598893 NCT03875729 NCT05757713	Hagopian et al. (2013) [28]	Reduced loss of C-peptide; maintained beta cell function; reduced insulin needs in this 2-year study.

Otelixizumab (TRX4)	Anti-CD3 monoclonal antibody	• Headache • Gastrointestinal issues • Arthralgia • Myalgia	NCT00678886 NCT01123083	Aronson et al. (2014) [40] Keymeulen et al. (2005) [35]	No change in C-peptide levels; no improvement in markers of metabolic control.

Daclizumab (Zinbryta, Zenapax)	Anti-CD25 monoclonal antibody	• Gastrointestinal infections • Neutropenia and leukopenia • Elevated liver enzymes • Hypoglycemia	NCT00064714 NCT00468117	Rother et al. (2009) [46]	No improvement in beta cell function.

Ladarixin	Inhibitor of IL-8 receptors (CXCR1 and CXCR2)	• Gastrointestinal infections • Dyspepsia • Headache	NCT04628481	None	N/A

Antithymocyte globulin (ATG) (Thymoglobulin, Atgam)	T lymphocyte depletion	• Fever • Headache • Nausea • Lymphopenia • Serum sickness	NCT01106157 NCT02215200 NCT00434811 NCT00468117	Haller et al. (2015) [65] Foster et al. (2018) [68]	Preserved beta cell function in patients with T1D for 4–24 months; lowered HbA1c; preserved T regulatory cells.

**Table 2:** Immunomodulators used to prevent islet transplant failure, tissue rejection, and cell death.

**Drug name(s): transplantation**	**Target**	**Side effects**	**NCT number(s)**
Alemtuzumab (Campath, Lemtrada)	Anti-CD52 monoclonal antibody	• Secondary autoimmune disorder	NCT00331162

Basiliximab (Simulect)	IL-2 receptor antagonist	• Constipation • Nausea • Headache • Hypophosphatemia • Hyperkalemia • Hypercholesterolemia • Anemia • Hypertension	NCT00434811 NCT00679042

Etanercept	Tumor necrosis factor alpha and beta inhibitor	• Risk of multiple sclerosis • Seizures • Lymphoma • Blood disorders	NCT00434811 NCT00468117

Reparixin	CXCR1 and CXCR2 inhibitor	• Risk of blood and lymphatic system disorders • Metabolic disorders • Musculoskeletal, connective tissue and nervous system disorders	NCT01220856 NCT01817959

## Data Availability

All data and methodologies will be made available upon request.
